# Environmental Epidemiology of Intestinal Schistosomiasis in Uganda: Population Dynamics of *Biomphalaria* (Gastropoda: Planorbidae) in Lake Albert and Lake Victoria with Observations on Natural Infections with Digenetic Trematodes

**DOI:** 10.1155/2015/717261

**Published:** 2015-02-01

**Authors:** Candia Rowel, Besigye Fred, Martha Betson, Jose C. Sousa-Figueiredo, Narcis B. Kabatereine, J. Russell Stothard

**Affiliations:** ^1^Vector Control Division, Ministry of Health, 15 Bombo Road, P.O. Box 1661, Kampala, Uganda; ^2^Royal Veterinary College, Hawkshead Lane, North Mymms, Hatfield, Hertfordshire AL9 7TA, UK; ^3^Centro de Investigação em Saúde de Angola, Hospital Provincial, Rua Direita do Caxito, Bengo, Angola; ^4^Department of Life Sciences, Natural History Museum, Wolfson Wellcome Biomedical Laboratories, Cromwell Road, London SW7 5BD, UK; ^5^Department of Parasitology, Liverpool School of Tropical Medicine, Pembroke Place, Liverpool L3 5QA, UK

## Abstract

This study documented the population dynamics of *Biomphalaria* and associated natural infections with digenetic trematodes, along the shores of Lake Albert and Lake Victoria, recording local physicochemical factors. Over a two-and-a-half-year study period with monthly sampling, physicochemical factors were measured at 12 survey sites and all freshwater snails were collected. Retained *Biomphalaria* were subsequently monitored in laboratory aquaria for shedding trematode cercariae, which were classified as either human infective (*Schistosoma mansoni*) or nonhuman infective. The population dynamics of *Biomphalaria* differed by location and by lake and had positive relationship with pH (*P* < 0.001) in both lakes and negative relationship with conductivity (*P* = 0.04) in Lake Albert. Of the *Biomphalaria* collected in Lake Albert (*N* = 6,183), 8.9% were infected with digenetic trematodes of which 15.8% were shedding *S. mansoni* cercariae and 84.2% with nonhuman infective cercariae. In Lake Victoria, 2.1% of collected *Biomphalaria*  (*N* = 13,172) were infected with digenetic trematodes with 13.9% shedding *S. mansoni* cercariae, 85.7% shedding nonhuman infective cercariae, and 0.4% of infected snails shedding both types of cercariae. Upon morphological identification, species of *Biomphalaria* infected included *B. sudanica*, *B. pfeifferi*, and *B. stanleyi* in Lake Albert and *B. sudanica*, *B. pfeifferi*, and *B. choanomphala* in Lake Victoria. The study found the physicochemical factors that influenced *Biomphalaria* population and infections. The number and extent of snails shedding *S. mansoni* cercariae illustrate the high risk of transmission within these lake settings. For better control of this disease, greater effort should be placed on reducing environmental contamination by improvement of local water sanitation and hygiene.

## 1. Introduction

Digenetic trematodes of the genus* Schistosoma* are the causative agents of schistosomiasis in man and have an indirect life cycle with free swimming larval stages: miracidia that infect freshwater gastropod snail hosts and cercariae, shed from infected snails, that enter mammalian hosts by direct penetration across intact skin. Infection with* S. mansoni* gives rise to intestinal schistosomiasis and is transmitted by several species within the genus* Biomphalaria* [1]. In Uganda, the distribution of* Biomphalaria *is countrywide although certain species may be restricted; for example, in the basin of Lake Victoria,* B. sudanica, B. choanomphala*, and* B. pfeifferi *are found, whereas in Lake Albert* B. sudanica, B. stanleyi*, and* B. pfeifferi* are the predominate species [1]. This interpretation was revised upon molecular characterization of sampled snails by detection of* B. choanomphala*, as largely indistinguishable upon shell morphology with local* B. stanleyi *[2]. Typically,* Biomphalaria* generally prefers stagnant or slow moving water with low wave action and shows a high degree of tolerance to variation in the temperature of its habitat [3]. Such conditions, though not always, may also prove favorable for hatched miracidia to find and locate snails and in so doing establish patent infections that subsequently produce cercariae thereby fulfilling an essential epidemiological requirement [4].

Owing to an intricate host-parasite evolutionary interplay, trematodes are highly specific for their mollusc host and miracidia of* S. mansoni* only develop successfully within* Biomphalaria* [5]. Schistosome miracidia remain infective for about 8–12 hours after hatching and, even if in nonflowing water, can disperse over distances of at least 5 m from the source of origin [6]. After the miracidium penetrates the epithelium of the snail, successful establishment depends on mitigating molluscan immunity which is both cellular and humoral [5, 7, 8]. The miracidium develops into primary sporocysts and secondary sporocysts that thence liberate cercariae, being shed from the snail typically bursting host tissues. Under favourable conditions, this prepatent period or cercarial incubation period lasts for a minimum of approximately three weeks and varies widely according to the host/parasite combination and environmental factors, especially temperature (reviewed by [9–14]). Once patent, an individual snail may shed cercariae for several months, more or less continuously [15]. As these larval stages often have unique morphological features and characteristics, identification of cercariae can be accomplished by visual examination under the dissecting microscope, timing of cercarial emergence, and their swimming characteristics [16, 17]. Owing to this incubation period, if a field caught snail is not presently shedding cercariae, it may be incubating an infection which becomes later patent some 3-4 weeks later.

*Biomphalaria *are generally tolerant over wide ranges of the commonly measured chemical and physical variables in fresh waters; however, increased saline concentration is an important factor in preventing schistosomiasis transmission [1]. Although the snail hosts of schistosomes tolerate a wide range of environmental conditions, their local distribution may be patchy, often showing marked aggregations along short distances of the margin of a lake or stream. Patchiness in snail distribution reflects factors such as protection from water flow, wave action, and desiccation, availability of food, presence of vegetation, and suitable surfaces for attachment [1, 14]. Spatial variation in the prevalence of patent snail infection is related, essentially, to the force of infection; that is, supply of miracidia derived from human excreta (for* S. mansoni*) which can also induce local snail mortality if infected by multiple miracidia. The chances that infection will develop to patency within an individual snail depend on genetic constitution, age, and length of prepatent period compared with snail life expectancy. This is equally true for infections in snails caused zoonotic schistosome species or by other trematodes that infect vertebrates other than man [18–20]. Many studies, however, have observed surprisingly low prevalences of patent snail infections in known schistosomiasis transmission sites [1, 13, 21], whilst increased abundance of* Biomphalaria* and transmission of* S. mansoni* in other sites are perhaps consequences of the construction of antisalinity barrage, engineering works which impede tidal flow [22–25] and environmental and climatic factors such as local geography, temperature, and rainfall [10–12, 26]. Some of these factors have been investigated at a local level in Lake Albert [27] and Lake Victoria [28], but as yet a detailed assessment through a longer monitoring period is lacking.

To shed light on the longer term patterns in the environmental epidemiology of intestinal schistosomiasis in Lake Victoria and Lake Albert in Uganda and compare and contrast settings, this study investigated the population dynamics of* Biomphalaria* and natural infections with trematodes, over a two-year study period and by assessing for relationships with local physicochemical factors.

## 2. Materials and Methods

### 2.1. Study Design and Area

**Figure 1:**
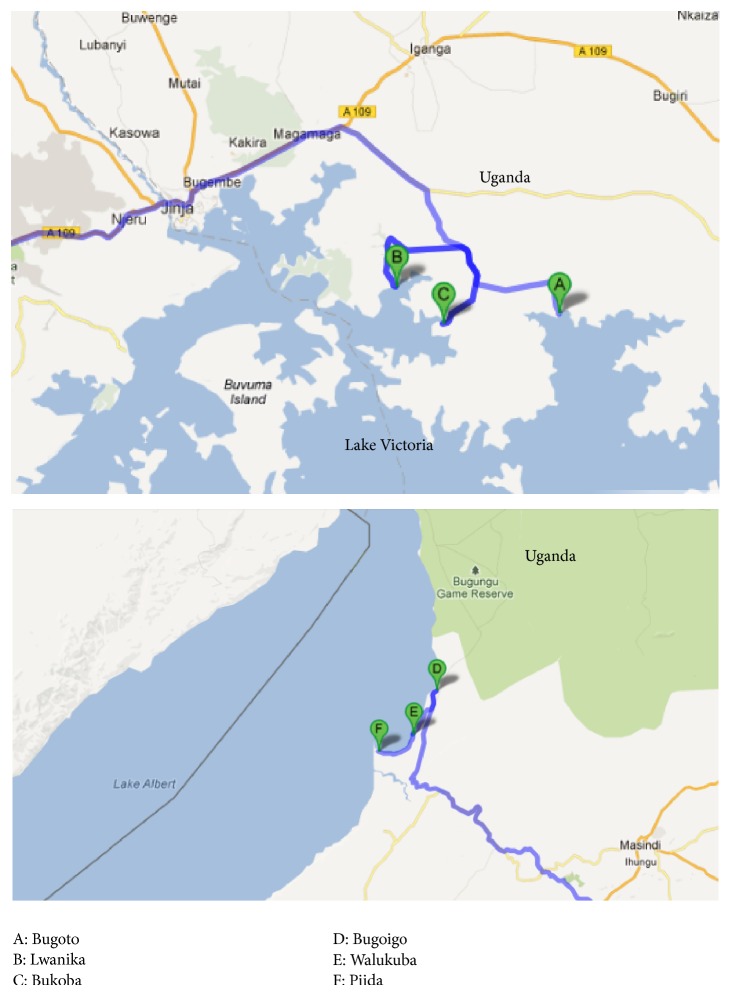
Map showing the locations of* Biomphalaria* snail collection along Lake Victoria and Lake Albert.

An observational and analytic study was conducted along the Ugandan shores of Lake Albert and Lake Victoria at six locations: Bugoigo (N01.90849° E031.40963° Elev.: 615 m), Walukuba (N01.84235° E031.37823° Elev.: 617 m), and Piida (N01.81950° E031.32818° Elev.: 618) in Buliisa District (Lake Albert) and Bugoto (N00.30900° E033.62154° Elev.: 1153 m), Lwanika (N00.35109° E033.446019° Elev.: 1128 m), and Bukoba (N00.31265° E033.49294° Elev.: 1133 m) in Mayuge District (Lake Victoria) (see Figure 1). At each site, snail collections were performed at the lake edge, which was often marshy, designated site A, and at depth (~1 m) in the lake as designated site B. The study was conducted from January 2009 to May 2011 and typically involved coordinated sampling at each site in a monthly timetable.

### 2.2. Study Site Selection and Sampling Protocol

The study was carried out as part of the Schistosomiasis in Mothers and Infants (SIMI) project, to sample snails at putative water contact sites where human activities like fetching, washing, and swimming took place (Figure 1). The aim was to later relate snail infection pattern to human infection with schistosomiasis. The sampled snails were sorted according to genera and the physicochemical factors were measured and observed.

### 2.3. Study Population

A total of 6,183* Biomphalaria *snails were collected along the shores of Lake Albert and 13,172* Biomphalaria* snails were collected along the shores of Lake Victoria in a period of 2 years and 5 months, with sampling done each month.

### 2.4. Data Collection Methods

#### 2.4.1. Scooping Method

The snail scoop net was used for scooping for 40 minutes in each location with 20 minutes in site A and 20 minutes in site B. The snails were put in a tray, sorted according to genus, thoroughly counted, and recorded on malacological form/proforma data sheets.

#### 2.4.2. Physicochemical Factors Measurement

A water meter (430 Enterprise; Jenway Ltd, Stone, UK) was used to measure water chemistry and physical parameter including conductivity, total dissolved solute, pH, and temperature. Water depth was measured using a calibrated water pole, Tinytag data loggers (Gemini Data Loggers, Chichester, UK) were also used to monitor the water temperature every 15 minutes, and observations on local wave actions, as made by eye, were used to assess wave exposure at the time of snail collection.

#### 2.4.3. Laboratory Investigations

After collection from the sampling sites, the* Biomphalaria* snails were kept alive in aquaria for four weeks. Each week the snails are checked for cercarial shedding: snails were individually put in shedding trays with mineral water, exposed to natural light for 2-3 hours, and examined under a dissecting microscope for presence or absence of cercariae. The cercariae were identified by the general anatomical appearance [29]. The shedding* Biomphalaria* snails were identified to species level using shell morphology [1]. All the information was recorded on proforma data sheets.

### 2.5. Quality Control

Prior to the commencement of the study, the equipment and materials were tested for accuracy and reliability by carrying out a pilot study. To prevent personnel bias, two technicians carried out the surveys, working interchangeably between Lake Albert and Lake Victoria in subsequent months. This was controlled for in the analyses and no significant effect of technician was observed (test statistic).

### 2.6. Data Handling and Statistical Analysis

Data on proforma data sheets were then transferred into electronic format using Microsoft Excel. The data collected were analyzed using STATA version 10.0.* Biomphalaria* infection prevalences were estimated and presented using tables and graphs. In order to determine physicochemical factors associated with abundance and infections of* Biomphalaria *snail at each Lake, a generalized linear model of Gaussian log function was applied. In this model, abundance and infections of snails were used as outcomes while independent variables included continuous variables, temperature, pH, conductivity, and total dissolved solute, and categorical variables, wave action and weather. Coefficient, *P* values, and 95% confidence interval (CI_95_) were estimated for physicochemical factors. *P* value ≤0.05 was a measure of significance level. The arithmetic mean was chosen as the measure of central tendency.

### 2.7. Ethical Consideration

The study protocol was reviewed and approved by the Ethical Review Committee of the Uganda National Council for Science & Technology (UNCST) and conducted within the framework of the ongoing Schistosomiasis Control Programme under the Vector Control Division (VCD), Ministry of Health.

## 3. Results

### 3.1. *Biomphalaria* Population Dynamics

**Figure 2:**
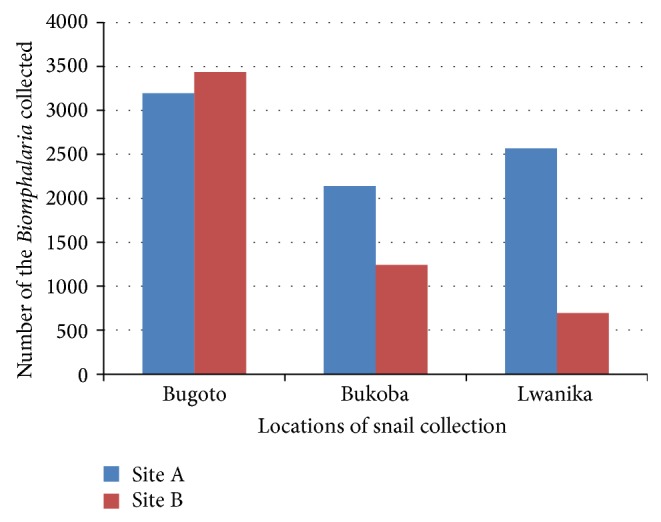
*Biomphalaria *population dynamics along Lake Victoria.

**Figure 3:**
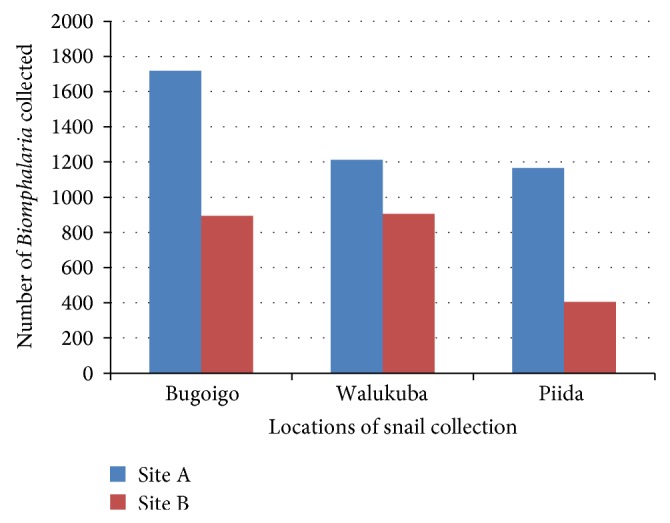
*Biomphalaria* population dynamics along Lake Albert.

There was variation in the number of* Biomphalaria *snails collected in different locations and sites despite the same period and duration of sampling used in all locations and sites. A total of 2,615 snails were sampled in Bugiogo, 2,081 in Walukuba, and 1,487 in Piida along the shores of Lake Albert (see Figure 3). In contrast, 6,530* Biomphalaria *were collected in Bugoto, 3,260 in Lwanika, and 3,382 in Bukoba along the shores of Lake Victoria (see Figure 2). This suggested that the* Biomphalaria *population was higher in Bugoigo and lower in Piida along Lake Albert and higher in Bugoto and lower in Lwanika along Lake Victoria. The* Biomphalaria *population also varied with site of snail collection in that the site A which was the shallow point had more* Biomphalaria *snails than site B which was a deeper point of snail collection along Lake Albert (see Figure 3). Along Lake Victoria, more* Biomphalaria *snails were found in site A than site B in Bukoba and Lwanika, whereas Bugoto had more* Biomphalaria *snails in site B than site A (see Figure 2).

### 3.2. The Physicochemical Factors

The physicochemical factors looked at included pH, temperature, conductivity, and total dissolved solute (TDS). These parameters varied greatly between the locations, sites, and the water bodies (see Table 1). Among the physicochemical factors measured, conductivity and the total dissolved solute (TDS) were significantly higher along Lake Albert than Lake Victoria.

### 3.3. Association of* Biomphalaria* Population Dynamics with Physicochemical Factors

The* Biomphalaria *population showed positive relationship with the pH (*P* > 0.001, CI_95_ 8.61–19.82) and negative relationship with the conductivity (*P* = 0.04, CI_95_ −0.46 to −0.02) along Lake Albert (see Table 2). However, along the shores of Lake Victoria, only pH showed a positive relationship with* Biomphalaria *population (*P* > 0.001, CI_95_ 15.60–27.68). Although TDS showed a positive relationship and conductivity showed a negative relationship along the shores of Lake Albert and Lake Victoria, respectively, they were not statistically significant (*P* = 0.10 and *P* = 0.37, resp.). The temperature did not show any relationship with the* Biomphalaria *population dynamics along the two water bodies.

### 3.4. *Biomphalaria* Infection Rate

In Lake Albert, 8.9% (551/6,183) of* Biomphalaria* were infected with trematodes of which 15.8% (87/551) were* S. mansoni *and 84.2% (464/551) were nonhuman infective species (see Table 3). Along the shores of Lake Victoria, 2.1% (280/13,172) of snails were infected, with 13.9% (39/280) shedding* S. mansoni* cercariae, 85.7% (240/280) shedding nonhuman infective cercariae, and 0.4% (1/280) shedding multiple infections (both human infective and nonhuman infective cercariae) (see Table 4). The multiple infections were found in Bukoba and in the* B. pfeifferi *species.

The* Biomphalaria* infections were found in species* B. sudanica*,* B. stanleyi*,* B. pfeifferi*, and* B. choanomphala*, where* B. sudanica*,* B. stanleyi*, and* B. pfeifferi* were found along Lake Albert, and* B. choanomphala*,* B. sudanica*, and* B. pfeifferi* were found along Lake Victoria. The species most affected by both types of cercariae (human infective and nonhuman infective cercariae) were* B. stanleyi* along Lake Albert and* B. pfeifferi* along Lake Victoria. The most affected species with* S. mansoni* cercariae were* B. stanleyi* along Lake Albert and* B. choanomphala* along Lake Victoria. Walukuba and Bugoto, along Lake Albert and Lake Victoria, respectively, were the locations in which most of* Biomphalaria* infected with human infective cercariae were found (see Tables 3 and 4).

### 3.5. *Biomphalaria* Infections over Time

**Figure 4:**
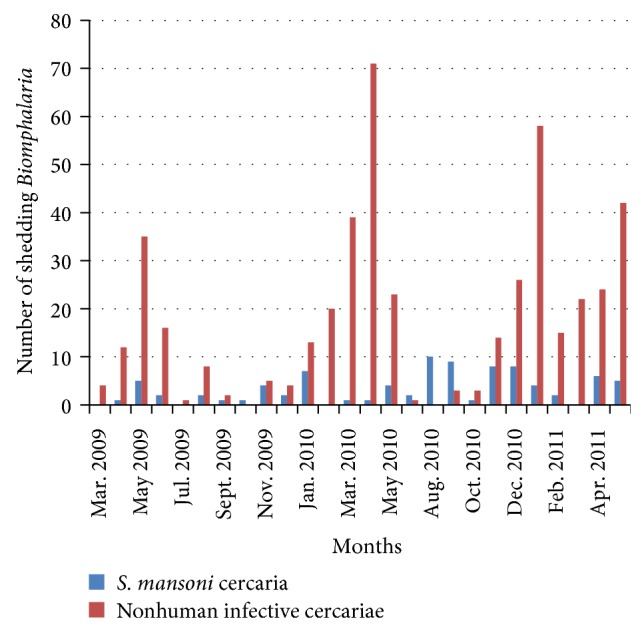
Temporal dynamics of* Biomphalaria* infections along Lake Albert.

**Figure 5:**
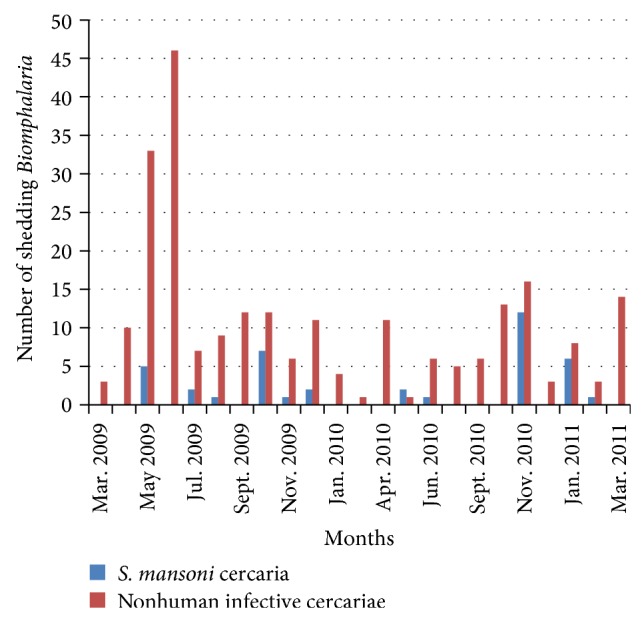
Temporal dynamics of* Biomphalaria *infections along Lake Victoria.

The* Biomphalaria *shedding cercariae were found throughout the months of the year.* Biomphalaria* shedding* S. mansoni *cercariae (human infective cercariae) were found in 22 months out of 26 months of the survey along Lake Albert and 11 months out of 24 months of survey along Lake Victoria (see Figures 4 and 5).* Biomphalaria* shedding nonhuman infective cercariae were found in 24 months out of 26 months of the survey along Lake Albert and all 24 months of Lake Victoria survey.

The fact that* Biomphalaria* shedding* S. mansoni *cercariae were found in most months of the year indicates continuous transmission of* S. mansoni*.

### 3.6. Factors Influencing* Biomphalaria* Infections

The* Biomphalaria* shedding* S. mansoni* cercariae had a positive relationship with* Biomphalaria *populations (*P* = 0.01, CI_95_ 0.002–0.014), a negative relationship with conductivity (*P* = 0.05, CI_95_ −0.003 to −0.001), and a positive relationship with temperature (*P* = 0.02, CI_95_ 0.011–0.123) along Lake Albert (see Table 5). A negative relationship with pH (*P* = 0.05, CI_95_ −0.44 to −0.003), a positive relationship with temperature (*P* = 0.03, CI_95_ 0.008–0.130), and a positive relationship with slight wave action (*P* = 0.03, CI_95_ 0.076–1.278) along Lake Victoria.

The* Biomphalaria* shedding nonhuman infective cercariae showed a positive relationship with temperature along Lake Albert (*P* = 0.008, CI_95_ 0.111–0.706) and Lake Victoria (*P* = 0.004, CI_95_ 0.037–0.182) (see Table 6). However, there was a negative relationship with conductivity which was not statistically significant (*P* = 0.059), while the rest of the factors did not show any relationship with the* Biomphalaria* shedding nonhuman infective cercariae.

**Figure 6:**
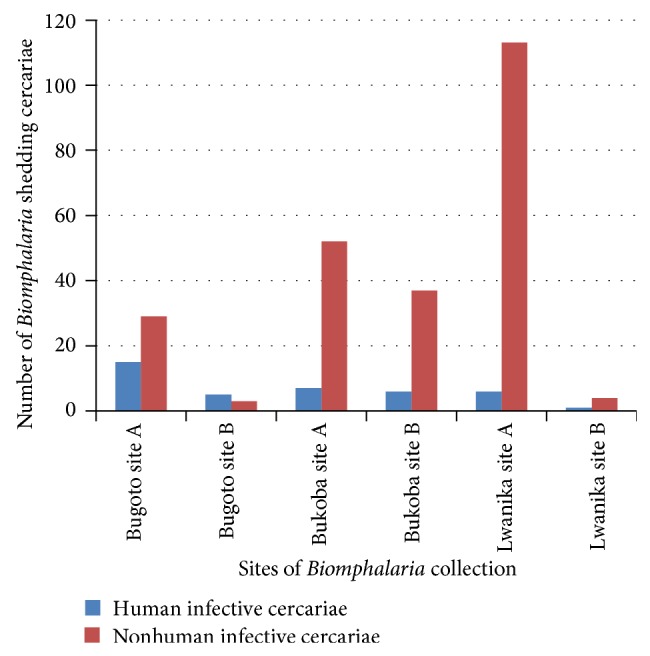
*Biomphalaria *shedding along Lake Victoria.

**Figure 7:**
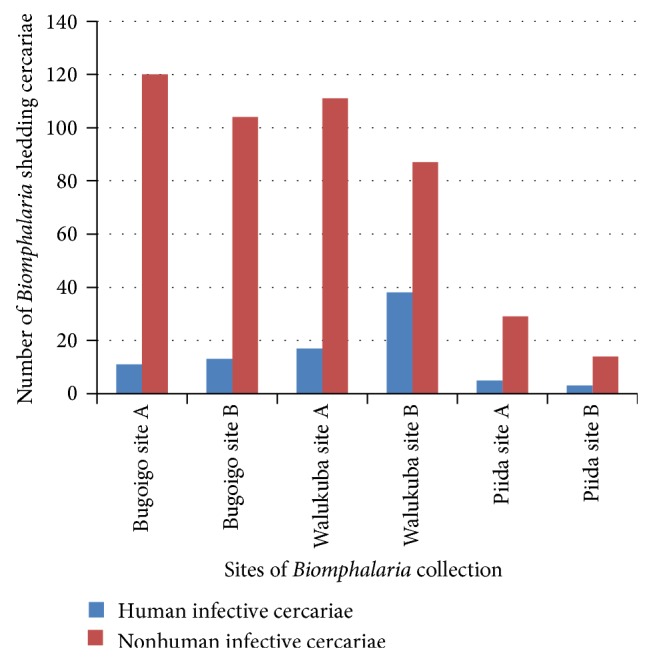
*Biomphalaria *shedding along Lake Albert.

The water depth in which the* Biomphalaria* was found seemed to influence its infection where, along Lake Victoria, shallow site A had more* Biomphalaria* shedding cercariae types than the deeper site B (see Figure 6). Along Lake Albert, the same situation was observed with nonhuman infective cercariae with more* Biomphalaria* infection in site A than site B (see Figure 7), whereas it was not the case with* Biomphalaria* shedding human infective cercariae which had varied abundance in sites in different locations.

## 4. Discussion

This study evaluated the physicochemical factors, which affect the* Biomphalaria* population dynamics and infection rates with trematode in its natural habitats along the Ugandan shores of Lake Albert and Lake Victoria. Like the associations presented by Standley et al. [28] and Levitz et al. [27], our results showed that some physicochemical factors had limited influence on* Biomphalaria *populations and associated trematode infections along the two water bodies. According to morphology, the most infected* Biomphalaria *species which shed* S. mansoni* cercariae were* Biomphalaria stanleyi *and* Biomphalaria choanomphala*; thus their control could significantly reduce* S. mansoni *transmission. Shedding was observed across most months of year, maintaining transmission throughout; therefore, to achieve maximum control of schistosomiasis, we recommend that greater effort should be placed on reducing environmental contamination by improvement of local water sanitation and hygiene.

### 4.1. *Biomphalaria* Population Dynamics and Variation in Physicochemical Factors

The* Biomphalaria* snail collection was done in every location within the same period and duration. The total number of* Biomphalaria* snails collected in different locations and sites varied. This was found to be attributed to conductivity along Lake Albert, which varied between locations and sites. There was a negative relationship of conductivity with the* Biomphalaria *populations (*P* = 0.04) along the shores of Lake Albert which corresponds with other studies done along the same lake [30]. This was evident in Piida where conductivity values were the highest (Table 1), hence fewer* Biomphalaria* snails collected (Figure 3). pH was also shown to influence the* Biomphalaria *populations in the water bodies and showed a positive relationship with* Biomphalaria* populations (*P* < 0.001) (Table 2).

The differences in effect of some physicochemical factors on* Biomphalaria* populations in the two water bodies could be due to different locations in which the two water bodies are found [31–34], which allowed considerable difference in the values of the physicochemical factors of the water bodies. The site of the* Biomphalaria *collection seemed to determine its abundance, especially along Lake Albert and some locations along Lake Victoria, where the shallow site A had higher* Biomphalaria* population than deeper site B (Figures 2 and 3). This could be because of a greater availability of the food for the* Biomphalaria* in the shallow site A than in deeper site B [35].

### 4.2. *Biomphalaria* Infection Rate Status

Both the human infective cercariae (*S. mansoni *cercariae) and nonhuman infective cercariae were shed by the* Biomphalaria *in all locations and sites. The snails showed surprisingly low* S. mansoni* infection prevalences despite the fact that the locations of snail collection were hyperendemic for* S. mansoni *(Tables 3 and 4). The low prevalence of* S. mansoni *cercariae reported here was in line with other studies [13, 21]. It is likely that the use of molecular methods would lead to detection of a higher prevalence of snails infected with* S. mansoni* parasites [36].

The prevalence of coinfections of* S. mansoni *and nonhuman infective trematodes was low (0.4%). This could be attributed to the physiological changes initiated by established trematodes in snails which can kill other invading trematodes or boost immune responses, a phenomenon termed behavioral fever [37–40]. This fact could be the explanation for the low* Biomphalaria *shedding human infective cercariae; for example, in the months in which more* Biomphalaria *were shedding nonhuman infective cercariae, there were fewer or no* Biomphalaria *shedding human infective cercariae (Figures 4 and 5). The* Biomphalaria *species involved in multiple infections was* B. pfeifferi* which corresponded with the species involved in Kenya [41] but contrasts with the species involved in multiple infections in Egypt, which was* B. sudanica* [41]. This could be because the surveys were carried along the same lake in the present study and in Kenya (Lake Victoria), which had similar environmental conditions which might have favoured coinfections in this particular species, whereas different water bodies were surveyed in Egypt.

The* Biomphalaria* species infected included* B. sudanica*,* B. pfeifferi*, and* B. stanleyi *along Lake Albert and* B. sudanica*,* B. pfeifferi*,and* B. choanomphala* along Lake Victoria. The presence of* B. pfeifferi* along Lake Albert has been doubtful, despite some studies reporting that snails closely resembling* B. pfeifferi* have been found in swamp areas of Lake Albert, Butiaba [42]. In additional studies to identify snails using molecular inferences and morphometry, of 18 specimens only a single specimen was identified as* B. pfeifferi* in Lake Albert [43]. These reports point to the potential presence of* B. pfeifferi *along Lake Albert; however, further confirmatory studies are required.

The* S. mansoni* cercariae prevalence was highest in Walukuba and Bugoto along Lake Albert and Lake Victoria, respectively, which hold 50% or more of the infected snails (Tables 3 and 4). However, previous studies done along Lake Albert in the same locations showed different results, where snails from Piida and Bugoigo had the highest schistosome infection rates [44]. This could mean that transmission rates at locations depend on human activities at each location which can change over time. The most commonly infected* Biomphalaria* species in this study were* B. stanleyi *and* B. choanomphala *along Lake Albert and Lake Victoria, respectively, where the finding along Lake Albert agreed with previous studies from Lake Albert which showed infection prevalence to be significantly higher in* B. stanleyi* [44, 45] and report along Lake Victoria indicated* B. choanomphala *a most efficient vector [46]. This information could be utilized to specifically target controlling these species in the water bodies to help in the control of schistosomiasis in these areas.

### 4.3. *Biomphalaria* Infection Patterns

The* Biomphalaria *snails shed nonhuman infective cercariae and* S. mansoni *cercariae. Both types of cercariae were shed in most months of the surveys in both lakes (Figures 4 and 5). This revelation casts doubt on the effectiveness of mass drug administration for schistosomiasis which is done once a year in these areas. As reinfection of people is inevitable, there is a need to rethink the strategy for schistosomiasis control in these areas which could prevent snail infection like sanitation improvement. The* Biomphalaria *shedding was found more in site A than site B along Lake Victoria and some location of Lake Albert (Figures 5 and 6). This fact could also be used in control strategy of schistosomiasis in that intensive snail control done in shallow margin of the lake, which would not have serious effect on ecosystem of the lakes but help in control of schistosomiasis.

### 4.4. Factors Influencing* Biomphalaria* Shedding

The* Biomphalaria* shedding* S. mansoni *cercariae showed a positive relationship with* Biomphalaria* population size (*P* = 0.01, CI_95_ 0.002–0.014) along Lake Albert. This was probably due to the fact that a higher population density of snails could promote parasite transmission as the access distance for the miracidia is reduced; hence more snails get infected, which could be the reason as to why the location with the lowest* Biomphalaria *abundance had least* Biomphalaria* infections (Figure 3 and Table 3). There was a negative relationship with conductivity (*P* = 0.05, CI_95_ −0.003 to −0.001) along Lake Albert. This could explain the low* Biomphalaria *infection along Lake Albert in the location with the highest conductivity values (Piida) (Figure 7). Temperature showed a positive relationship with* S. mansoni* cercarial shedding (*P* = 0.02, CI_95_ 0.011–0.123) along Lake Albert. This could be attributed to the favourable temperature at this location (arithmetic mean 27°C–28.7°C) which could have influenced the development and survival of the intramolluscan stages of schistosomes [10–12].

Along Lake Victoria there was negative relationship of the* Biomphalaria* snails shedding* S. mansoni* cercariae with pH (*P* = 0.05, CI_95_ −0.440 to −0.003), but, considering the pH values in the two water bodies, there was not much difference (Table 1) which could mean the effect was influenced by other factors along this water body [47]; for example, the pH and temperature could have effect on the physiology and maturation rates of the parasite in the vector [48]. Temperature had a positive relationship with shedding (*P* = 0.03, CI_95_ 0.008–0.130) which could be attributed to the temperature range (Table 1) that influenced the development and survival of the intramolluscan stages of the cercariae [10–12]. In addition, slight wave action showed a positive relationship (*P* = 0.03, CI_95_ 0.076–1.278) (Table 5), which could be as a result of the increased contact between the snails and the miracidia that infect the snails more in a water body with slight wave action than in calm water. However, the difference in effect of some of the physicochemical factors along Lake Albert and Lake Victoria could be the fact that they are in different water bodies in which these factors exert varied effect [31–34].

Meanwhile, the* Biomphalaria* shedding nonhuman infective cercariae had positive relationship with temperature in both Lake Albert and Lake Victoria (*P* = 0.008, CI_95_ 0.111–0.706 and *P* = 0.04, CI_95_ 0.037–0.182). This could be because the temperature provided favourable condition which could have influenced the development and survival of the intramolluscan stages of the cercariae [10–12].

## 5. Conclusion

This study revealed that the physicochemical factors influencing the* Biomphalaria *population were conductivity, water depth, and pH, whereas those that influenced* Biomphalaria *infections were temperature, pH, slight wave action, water depth, conductivity, and* Biomphalaria *population density. Both human infective cercariae and nonhuman infective cercariae were shed by* Biomphalaria*. The shedding spread across most of the months of year, hence maintaining transmission throughout the year; therefore, in order to achieve maximum control of schistosomiasis, we recommend greater effort should be placed on reducing environmental contamination by improvement of local water sanitation and hygiene.

## Highlights

- Investigation into the effect of physicochemical factors on* Biomphalaria *population dynamics.
- Investigation into the effect of physicochemical factors on* Biomphalaria *infection in its natural habitat.
- Differentiating the cercariae types into human infecting and nonhuman infecting types, based on pattern of emergence and morphological appearance.
- Establishment of* Biomphalaria *species infected and locations in which they are found.

## Figures and Tables

**Table 1:** The arithmetic mean of the physicochemical factors of water along Lake Albert and Lake Victoria.

Sites of sample collection	Arithmetic mean			
	pH	Conductivity (*μ*s)	Total dissolved solute (ppm)	Temperature (°C)
Bugoigo				
Site A	8.38	619	398	28.7
Site B	8.07	740	347	28.5
Walukuba				
Site A	8.86	660	373	28.2
Site B	8.60	704	348	28.0
Piida				
Site A	7.72	999	568	27.8
Site B	7.68	1081	516	27
Bugoto				
Site A	7.69	270	148	27.3
Site B	8.62	112	58	27.7
Bukoba				
Site A	7.48	130	148	30.4
Site B	7.43	132	58	29.1
Lwanika				
Site A	9.22	161	89	29.0
Site B	8.53	165	62	28.6

**Table 2:** Generalized linear model (GLM) of *Biomphalaria* population density with physicochemical factors of water.

	Coefficient	*P* > *t*	[95% confidence interval, CI_95_]
Lake Albert			
pH	14.22	**<0.001**	8.61, 19.82
Conductivity	−0.24	**0.04**	−0.46, −0.02
Lake Victoria		** **	
pH	21.64	**<0.001**	15.60, 27.68

**Table 3:** *Biomphalaria* infections along Lake Albert.

Type of cercariae	Proportion of infected *Biomphalaria*	Species infected			Location affected		
		*B. sudanica *	*B. pfeifferi *	*B. stanleyi *	Bugoigo	Walukuba	Piida
Human infective cercariae (*S. mansoni* cercariae)	15.8% (87/551)	25.3% (22/87)	5.7% (5/87)	69% (60/87)	27.6% (24/87)	63.2% (55/87)	9.2% (8/87)

Nonhuman infective cercariae	84.2% (464/551)	45.5% (211/464)	11.6% (54/464)	42.9% (199/464)	47.6% (221/464)	43.1% (200/464)	9.3% (43/464)

**Table 4:** *Biomphalaria* infections along Lake Victoria.

Type of cercaria	Proportion of infected *Biomphalaria *	Species infected			Location affected		
		*B. sudanica *	*B. pfeifferi *	*B. choanomphala *	Bugoto	Lwanika	Bukoba
Human infective cercariae (*S. mansoni* cercariae)	13.9% (39/280)	22.5% (9/40)	20% (8/40)	57.5% (23/40)	50% (20/40)	17.5% (7/40)	32.5% (13/40)

Nonhuman infective cercariae	85.7% (240/280)	41.3% (99/240)	41.7% (100/240)	17.1% (41/240)	13.8% (33/240)	49.2% (118/240)	37.1% (89/240)

Human infective cercariae and nonhuman infective cercariae coinfections	0.4% (1/280)	0%	100% (1/1)	0%	0%	0%	100% (1/1)

**Table 5:** Generalized linear model (GLM) of *Biomphalaria* shedding *S. mansoni* cercaria with physicochemical factors of water and *Biomphalaria *population density along Lake Albert and Lake Victoria.

	Coefficient	*P* > *t*	[95% confidence interval, CI_95_]
L. Albert			
*Biomphalaria *population	0.01	**0.01**	0.002, 0.014
Conductivity	−0.002	**0.050**	−0.003, −0.001
Temperature	0.07	**0.02**	0.011, 0.123
Lake Victoria			
pH	−0.22	**0.05**	−0.440, −0.003
Temperature	0.07	**0.03**	0.008, 0.130
Wave action (ref. calm)			
Slight	0.68	**0.03**	0.076, 1.278

**Table 6:** Generalized linear model (GLM) of *Biomphalaria *shedding nonhuman infective cercariae with physicochemical factors of water and *Biomphalaria *population density along Lake Albert and Lake Victoria.

	Coefficient	*P* > *t*	[95% confidence interval, CI_95_]
L. Albert			
Temperature	0.409	**0.008**	0.111, 0.706
L. Victoria			
Temperature	0.110	**0.004**	0.037, 0.182
